# Modeling HIV Vaccines in Brazil: Assessing the Impact of a Future HIV Vaccine on Reducing New Infections, Mortality and Number of People Receiving ARV

**DOI:** 10.1371/journal.pone.0011736

**Published:** 2010-07-23

**Authors:** Maria Goretti P. Fonseca, Steven Forsythe, Alexandre Menezes, Shilpa Vuthoori, Cristina Possas, Valdiléa Veloso, Francisca de Fátima Lucena, John Stover

**Affiliations:** 1 Instituto de Pesquisa Clínica Evandro Chagas, Fundação Oswaldo Cruz, Rio de Janeiro, Rio de Janeiro, Brazil; 2 Futures Institute, Glastonbury, Connecticut, United States of America; 3 International AIDS Vaccine Initiative, New York, New York, United States of America; 4 Rabin Strategic Partners, New York, New York, United States of America; 5 Departamento de DST, AIDS e Hepatites Virais, Ministério da Saúde, Brasília, Federal District, Brazil; 6 Ministério do Desenvolvimento Social e Combate à Fome, Brasília, Federal District, Brazil; University of Cape Town, South Africa

## Abstract

**Background:**

The AIDS epidemic in Brazil remains concentrated in populations with high vulnerability to HIV infection, and the development of an HIV vaccine could make an important contribution to prevention. This study modeled the HIV epidemic and estimated the potential impact of an HIV vaccine on the number of new infections, deaths due to AIDS and the number of people receiving ARV treatment, under various scenarios.

**Methods and Findings:**

The historical HIV prevalence was modeled using Spectrum and projections were made from 2010 to 2050 to study the impact of an HIV vaccine with 40% to 70% efficacy, and 80% coverage of adult population, specific groups such as MSM, IDU, commercial sex workers and their partners, and 15 year olds. The possibility of disinhibition after vaccination, neglecting medium- and high-risk groups, and a disease-modifying vaccine were also considered. The number of new infections and deaths were reduced by 73% and 30%, respectively, by 2050, when 80% of adult population aged 15–49 was vaccinated with a 40% efficacy vaccine. Vaccinating medium- and high-risk groups reduced new infections by 52% and deaths by 21%. A vaccine with 70% efficacy produced a great decline in new infections and deaths. Neglecting medium- and high-risk population groups as well as disinhibition of vaccinated population reduced the impact or even increased the number of new infections. Disease-modifying vaccine also contributed to reducing AIDS deaths, the need for ART and new HIV infections.

**Conclusions:**

Even in a country with a concentrated epidemic and high levels of ARV coverage, such as Brazil, moderate efficacy vaccines as part of a comprehensive package of treatment and prevention could have a major impact on preventing new HIV infections and AIDS deaths, as well as reducing the number of people on ARV. Targeted vaccination strategies may be highly effective and cost-beneficial.

## Introduction

After 30 years the AIDS pandemic continues to compromise the health, quality of life and productivity of populations, and significantly affects economies as well as societal and family structures. Although the epidemic in Brazil remains concentrated amongst populations with increased vulnerability to HIV infection [1], more than 500,000 AIDS cases and 200,000 AIDS deaths have been reported by 2008 [2].

To address the challenge of continued HIV transmission as well as increasing treatment needs, the Brazilian government has prioritized prevention efforts [3]–[4], and has implemented a broad treatment and care program throughout the five geographical regions in the country. The treatment program is based on technical guidelines determined by a national expert committee, which ensures that all patients living with HIV and AIDS in need of antiretroviral (ARV) treatment have access to state-of-the-art care, including options for patients resistant to first and second line regimens [5].

While much has been achieved with the adoption of existing preventive measures and the introduction of potent drugs that severely reduce viral load, there is still a pressing need to develop additional prevention options, such as HIV vaccines, to drastically reduce the number of new infections. A number of studies have been published that involve the application of mathematical modeling to questions of the potential impact of an HIV vaccine on HIV incidence or prevalence in a variety of epidemic settings (see [6] for a review of this literature). These studies have generally shown that even a moderate-efficacy vaccine could have a significant impact in reducing new HIV infections and could represent a vital strategy to control the epidemic. Recent mathematical model studies have shown that a moderately effective vaccine given to the adult population could reduce future HIV prevalence in low- and middle- income countries by 30% to 35% in 10 to 15 years [7]–[8].

Considering the specificities of the AIDS epidemic in Brazil, this study was undertaken to model the Brazilian epidemic and to estimate the potential impact of an HIV vaccine on the number of new infections, deaths due to AIDS and the number of people receiving antiretroviral (ARV) treatment, under various scenarios.

## Methods

The Spectrum HIV Vaccine Module [9] was used to model the historical prevalence of HIV in Brazil from 1975 to 2010 and the impact of an HIV vaccine on the future course of the epidemic. Details of the model structure and key assumptions are available elsewhere [10]. The model divides the sexually active population aged 15–49 by sex and risk group. Although some infections occur at ages above 50, the percentage is small, less than 10%, and most behavioral indicators refer to the 15–49 age group. The risk groups are injecting drug users, men who have sex with men, high risk heterosexuals (female sex workers and their male clients), medium risk heterosexuals (men and women with more than one partner in the last year) and low risk heterosexuals (men and women with only one partner in the last year). The probability of acquiring a new HIV infection is determined by characteristics of the index person (number of partners), the partner (HIV status, stage of infection, ART use), and the partnership (sex acts per partner, condom use, STI prevalence, heterosexual or MSM contact, male circumcision status). Infected persons progress through a primary stage of infection with high infectivity, an asymptomatic stage with low infectivity, a symptomatic stage with high infectivity, to AIDS death. Infectivity is reduced by ARV use. The model includes a component to estimate the effects of prevention interventions on key behaviors (condom use, number of partners, unsafe injecting behavior) based on a summary of the impact literature [11].

The model was set up for Brazil using behavioral data from surveys [12]–[22], epidemiological [2], [23] and ART data [24] from the Brazilian Ministry of Health (MOH). We calibrated the model to HIV prevalence data by risk group and validated it by comparing the estimated AIDS mortality, HIV prevalence and the number of people receiving ARV with respective data from the MOH [2], [23]–[24]. Although there are no official estimates of incidence to which the model results can be compared, the number of new infections estimate is a consequence of matching official estimates of mortality and prevalence. The parameter [25]–[28] and behavioral values used [12]–[22] are shown in Tables 1 and 2.

**Table 1:** Parameter Values used to Simulate the Brazilian HIV Epidemic.

Parameter	Value	Source
Relative infectiousness compared to asymptomatic stage		Pilcher [25]
Primary stage	8	
Symptomatic stage	4	
Probability of HIV transmission per act, heterosexual contact	0.0007	Powers [26]
Transmission multiplier for MSM contacts	2.6	Vittinghoff [27]
Duration of (in years):		Fitted
Asymptomatic stage	10	
Symptomatic stage	3	
Condom efficacy	0.8	Weller [28]
Annual survival on ART	0.95	

**Table 2:** Current Behaviors used to Simulate the Brazilian HIV Epidemic, 2007.

	Risk groups					
	Not sexually active	Low	Medium	High	MSM	IDU
Population distribution (%)						
Males	6.5	66.5	18.2	4.5	3.5	0.8
Females	15.3	78.8	4.6	0.9	-	0.4
Condom use (%)		7	16	52	80	
Partners #						
Males		1	3	10	4	
Females		1	3	75	-	
Sex acts per partner #		70	40	5	25	

Source [12]–[22].

The HIV vaccine module can be used to examine the effects of vaccines with difference characteristics: reduction in susceptibility, disease progression and/or infectiousness, duration of effectiveness and take or degree type of action. Vaccination programs can be defined in terms of coverage over time of the entire adult population or targeted coverage for specific population groups.

The impacts of vaccines with different characteristics and the impacts of different implementation strategies were explored under a variety of different assumptions. In this paper, we present the results for a vaccine introduced in 2015 and with 80% coverage reached by 2020, with duration of 20 years. We assume that an HIV test is not a requirement for vaccination. ART coverage is nearly universal in Brazil today and we assumed that would continue in all projections.

The vaccine scenarios explored the potential impact of a 40% and 70% effective preventive “take” vaccine, which reduces both susceptibility and transmission. A “take” vaccine is one that achieves an efficacy of 50%, for example, by completely protecting 50% of those vaccinated and providing no protection for the other 50%, as opposed to a “degree” vaccine that achieves 50% efficacy by reducing susceptibility by 50% in all those vaccinated.

This study also looked at the potential impact of diverse vaccination strategies, including vaccinating the general adult population, aged 15–49 (including high risk populations); vaccinating 15-year-old adolescents; and vaccinating medium- and high-risk groups, defined as men who have sex with men (MSM), injecting drug users (IDU), and commercial sex workers and their partners (considered here as medium- and high-risk behavior heterosexuals).

In addition, a vaccine scenario that models the potential impact of a disease-modifying vaccine, defined as a vaccine that reduces infectiousness and delays progression to the development of AIDS, and consequently slows the progression to AIDS death, or, in other words, that would not affect individual susceptibility to HIV infection but instead reduces infectiousness and lengthens the amount of time spent in the asymptomatic phase by reducing viral load, was explored.

To assess the potential effect of behavioral disinhibition on the trajectory of Brazil's epidemic in the presence of an AIDS vaccine, vaccine impact scenarios were explored in which a 50% decline in condom use was considered either immediately after vaccine introduction or more gradually over time. To further explore the effectiveness of targeted vaccination strategies, the study also modeled the impact of failure to reach the medium- and high-risk population groups, by vaccinating 20% of these groups while reaching 80% of low-risk adult population aged 15–49.

## Results

**Figure 1:**
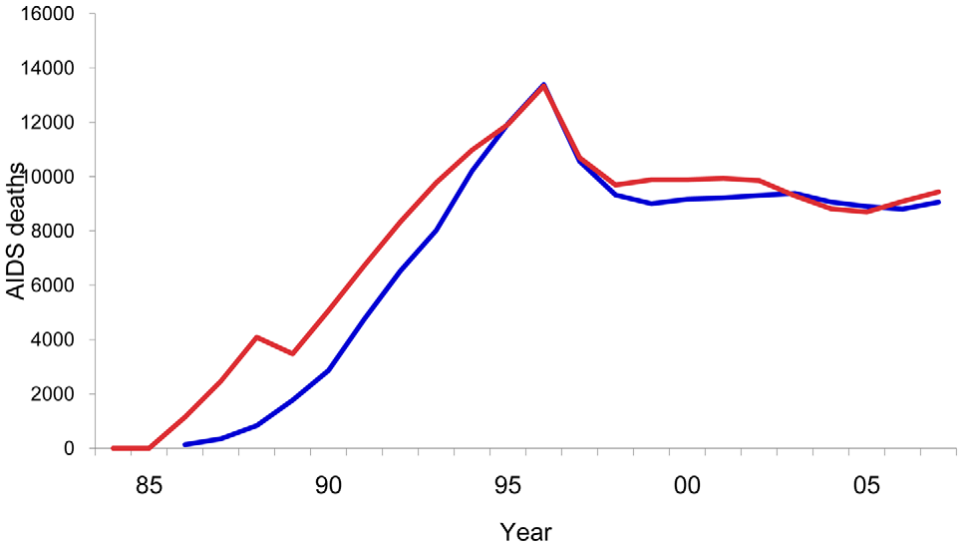
AIDS deaths model fit. Number of AIDS deaths and model fit. Brazil, 1985–2007. The blue line, deaths for age group 15–49 as reported by the Brazilian National Mortality Information System; the red line, Spectrum estimates of number of AIDS deaths in the same age group.

**Figure 2:**
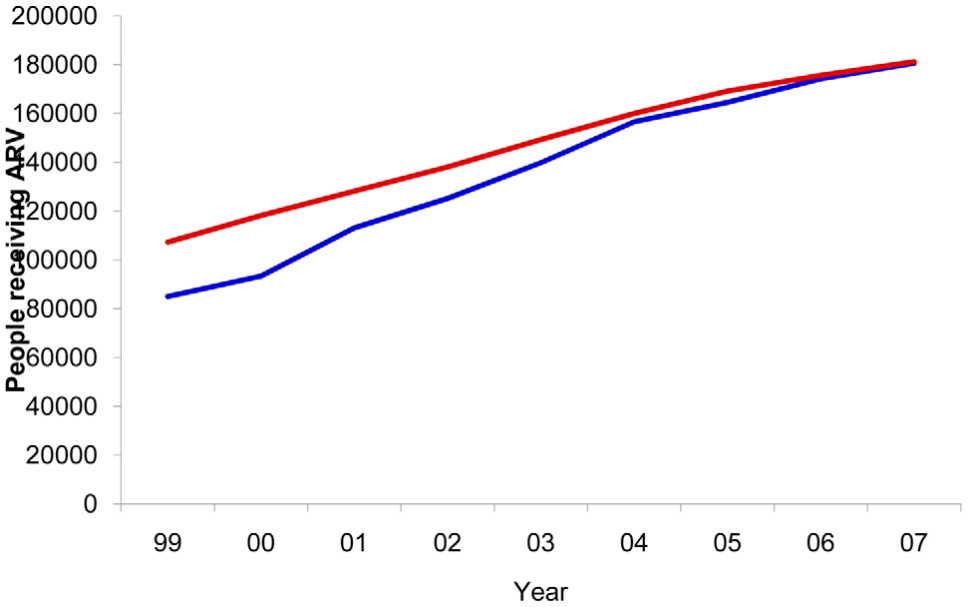
ARV patients and model fit. Number of patients receiving ARV and model fit. Brazil, 1999–2007. The blue line, number of patients of all age reported by the Brazilian STD, AIDS and Viral Hepatitis Department; the red line, Spectrum estimates of number of patients receiving ARV aged 15–49.

The model consistency can be seen in Figures 1 and 2. As can be observed, the progression of AIDS deaths over time adequately reflects the history of the epidemic in Brazil (Figure 1). Spectrum estimated 182,562 cumulative AIDS deaths for those aged 15–49, 12.3% more deaths when compared with 162,545 deaths reported by the Brazilian Mortality Information System for the period 1985–2007. When comparing the number of people receiving ARV, the Spectrum estimates were also similar to the official data, which report about 190,000 on ARV by the end of 2008 (Figure 2). The HIV prevalence estimated by Spectrum was slightly lower (0.40%) in 2004, when compared to a 68% confidence interval from the official estimate (0.44%–0.77%).

In order to capture the longer- term impact of an HIV vaccine, we simulated the epidemic to 2050. Spectrum projects 1.1 million new infections and 468,000 deaths from 2010 to 2050, keeping HIV prevalence relatively stable around 0.42 in the absence of a vaccine or changes in preventive measures and in patient treatment coverage.

### Scenarios to study the impact of an HIV vaccine in the Brazilian epidemic

**Figure 3:**
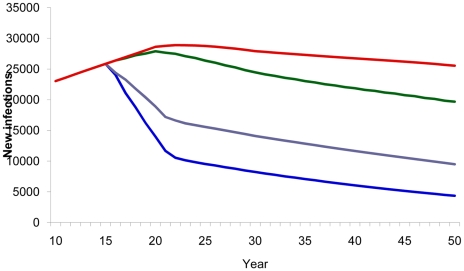
HIV vaccine impact on new infections. Number of estimated new HIV infections according to population vaccinated by year. Red line, baseline projection in the absence of a vaccine; green line, vaccination of adolescents (15 years old); purple line, vaccination of specific population: MSM, IDU, medium- and high-risk heterosexuals; blue line, vaccination of adults aged 15–49.

**Figure 4:**
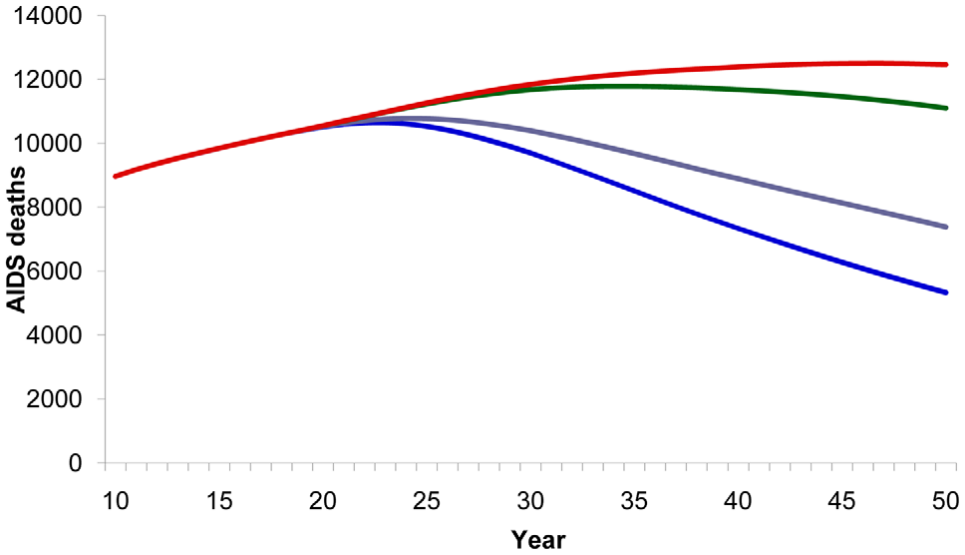
HIV vaccine impact on AIDS deaths. Number of estimated AIDS deaths according to population vaccinated by year. Red line, baseline projection in the absence of a vaccine; green line, vaccination of adolescents (15 years old); purple line, vaccination of specific population: MSM, IDU, medium- and high-risk heterosexuals; blue line, vaccination of adults aged 15–49.

Compared with a baseline scenario with no vaccine, vaccinating 80% of the adult population with a 40% efficacy vaccine reduced the cumulative number of new HIV infections from 2020–2050 by 73% (Figure 3) and AIDS deaths by 30% (Figure 4). Vaccinating the medium- and high-risk behavior population resulted in an estimated 52% reduction in new infections (Figure 3) and 21% reduction in AIDS deaths (Figure 4). However, the smallest impact was observed when vaccination strategies targeted only adolescents.

A 70% effective vaccine given to the general population would result in a significantly greater impact, interfering dramatically in the Brazilian epidemic by reducing the number of new infections from 2020 to 2050 by 92%. Even a targeted strategy of vaccinating only medium- and high-risk behavior population groups with a 70% effective vaccine would still produce dramatic results, reducing new infections by 74%.

### A disease-modifying vaccine scenario

**Figure 5:**
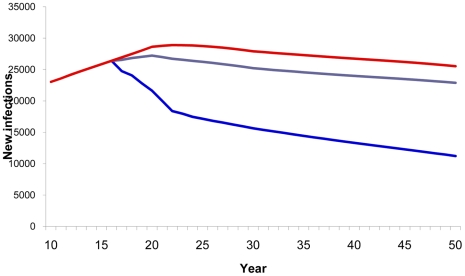
Impact of disease-modifying vaccine on number of new HIV infections. Number of new HIV infections according to population vaccinated with a disease-modifying vaccine. Red line, baseline projection in the absence of a vaccine; blue line, vaccination of adults aged 15–49; purple line, vaccination of specific population: MSM, IDU, medium- and high-risk heterosexuals.

**Figure 6:**
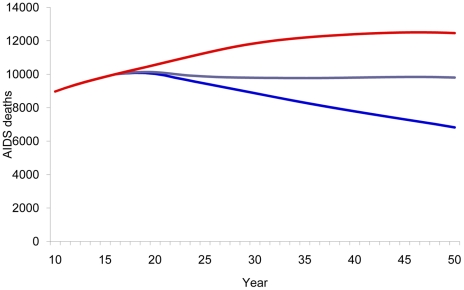
Impact of disease-modifying vaccine on number of AIDS deaths. Number of AIDS deaths according to population vaccinated with a disease-modifying vaccine. Red line, baseline projection in the absence of a vaccine; blue line, vaccination of adults aged 15–49; purple line, vaccination of specific population: MSM, IDU, medium- and high-risk heterosexuals.

When the general population was vaccinated with a disease-modifying vaccine, the cumulative number of new infections was reduced by 46% (Figure 5) and the number of deaths had a 30% reduction (Figure 6) from 2020 to 2050. A lower impact was observed when only specific groups were targeted for vaccination.

**Figure 7:**
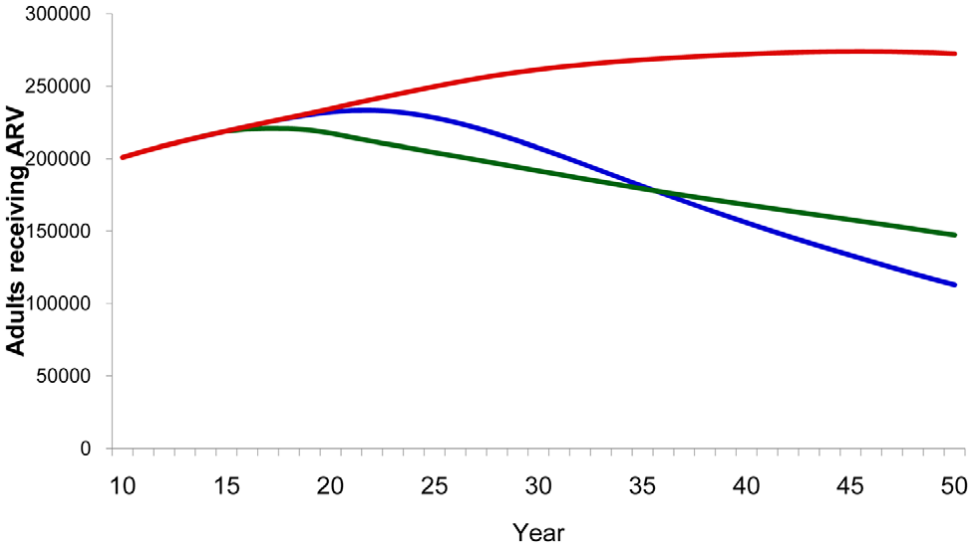
Patients on ARV. Estimated number of patients receiving ARV according to type of vaccine by year. Red line, baseline projection in the absence of a vaccine; blue line, 40% efficacy preventive vaccine; green line, 100% disease-modifying vaccine with 40% reduction in infectiousness.

Disease-modifying vaccines were also shown to be effective in reducing the number of patients receiving ARV, although not as effective as preventive vaccines (Figure 7).

### Assessment of the relative impact of targeted vaccination strategies

With the understanding that public health officials will consider vaccination strategies not only on the basis of their overall impact, but also taking into account dose availability at the time of licensing, cost of the vaccine and highest impact in relation to investment, the study explored how the different vaccine strategies compared in terms of impact and the number of doses required for each strategy. Table 3 summarizes the vaccine efficacy, the percentage reduction, the cumulative number of new HIV infections averted, the number of doses required and the number of HIV infections averted per 100,000 vaccinations, according to population vaccinated from 2020 to 2050, for both preventive and disease-modifying vaccines. With 80% coverage, the highest reduction in the number of new infections is reached if the general population aged 15–49 is vaccinated, although requiring a very high number of vaccine doses. On the other hand, the better result in terms of number of new infections averted per 100,000 doses is obtained when medium- and high-risk behavior population groups are prioritized for vaccination, with 4 times more infections averted compared to vaccinating the general adult population (Table 3).

**Table 3:** Vaccination Strategies.

Population to be vaccinated	New HIV infections (#)	Reduction in new infections (%)*	Infections averted (#)	Doses of vaccine required (per 1 million)	Infections averted per 100,000 vaccinations (#)
**Preventive Vaccine/Vaccine efficacy (%)**					
Adults (aged 15–49)					
40	230203	72.8	617647	241	256
70	72369	91.5	775481	241	322
Medium- & High-risk behavior groups					
40	405639	52.2	442211	43	1023
70	222754	73.7	625096	43	1447
15-year-olds					
40	725383	14.4	122467	43	283
70	643471	24.1	204379	43	472
Adults, excluding medium- and high- risk behavior groups					
40	408880	51.8	438970	208	211
70	214547	74.7	633303	208	304
**Disease-Modifying Vaccine**					
Adults (aged 15–49)	458773	45.9	389077	241	161
Medium- & High-risk behavior groups	767485	9.5	80365	43	186

Vaccine efficacy, cumulative number of new HIV infections, percentage reduction in relation to a no vaccine scenario, cumulative number of new HIV infections averted, number of doses required, and number of infections averted per 100,000 population vaccinated according to population to be vaccinated and type of vaccine (preventive or disease modifying).

*Compared to No Vaccine Scenario, where 847,850 new HIV infections would occur from 2020 to 2050 in the absence of vaccination.

### Behavior reversal (disinhibition) and failure to reach medium- and high-risk behavior population groups

**Figure 8:**
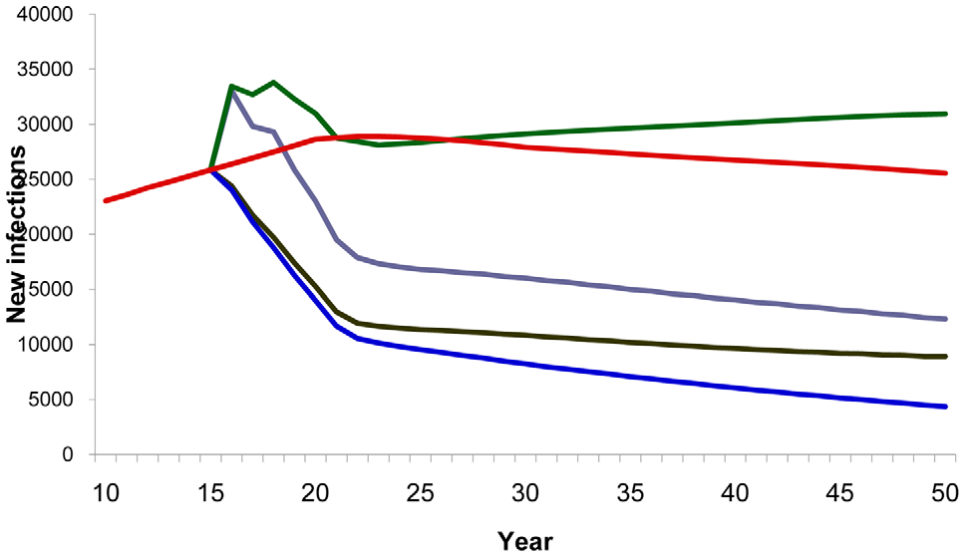
Impact of disinhibition with 40% efficacy vaccine. Number of new HIV infections estimates with a 40% efficacy vaccine and presence of disinhibition. Red line, baseline projection in the absence of a vaccine; blue line, vaccination of adults aged 15–49; brown line, vaccination of adults aged 15–49 with gradual disinhibition; purple line, vaccination of adults aged 15–49 with instantaneous disinhibition; green line, vaccination of specific population (MSM, IDU, medium- and high-risk heterosexuals) with instantaneous disinhibition.

**Figure 9:**
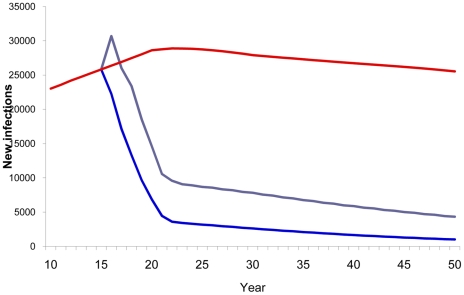
Impact of disinhibition with 70% efficacy vaccine. Number of new HIV infections estimates with a 70% efficacy vaccine and presence of disinhibition. Red line, baseline projection in the absence of a vaccine; blue line, vaccination of adults aged 15–49; purple line, vaccination of adults aged 15–49 with instantaneous disinhibition.

A few additional scenarios were considered to assess the relevance of factors that may reduce or interfere with the impact of a vaccine, such as lack of effective education and counseling of vaccine recipients, leading to behavior disinhibition, and failure to reach and vaccinate populations at higher risk. Two scenarios were considered to look into the potential importance of behavior disinhibition. In the first scenario, the number of new infections was reduced by 42% (42% instead of 73%) when the general population was vaccinated with a low-efficacy vaccine and gradually reduced condom use. In the second analysis regarding behavior dishinibition, the study observed an 8.5% increase in the cumulated number of new HIV infections when 50% of the medium- and high-risk behavior vaccinated population immediately stopped using condoms (Figure 8). This remains true even with a more effective vaccine (Figure 9).

**Figure 10:**
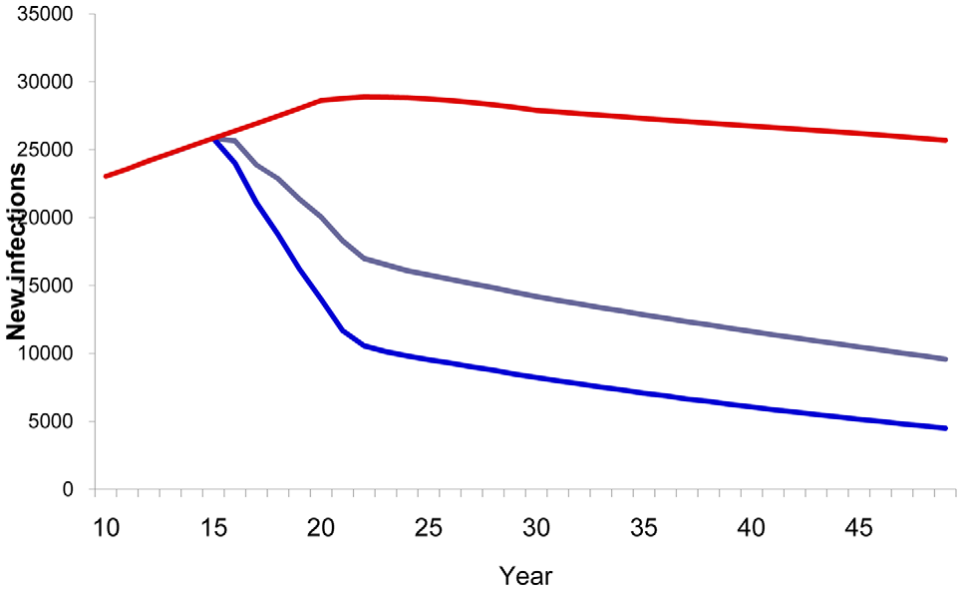
Failure to reach medium- and high-risk behavior groups. Estimated number of new HIV infections with a 40% efficacy vaccine according to vaccine coverage (%) of population targeted. Red line, baseline projection in the absence of a vaccine; blue line, 80% coverage of adults aged 15–49; purple line, 80% coverage of low-risk-adults aged 15–49 and 20% coverage of specific population: MSM, IDU, medium- and high-risk heterosexuals.

The analysis shows that the most effective strategies are those that target vaccinations to medium- and high-risk groups, as seen by the number of infections averted per 100,000 people vaccinated in Table 3. To demonstrate the importance of ensuring adequate coverage of high-risk individuals, the study explored the impact of reaching 80% vaccination coverage among the low-risk adult population, but only including 20% of the medium- and high-risk behavior groups in the vaccinated cohort. This scenario was shown to prevent 29% fewer new HIV infections (52% compared to 73%) when compared to a vaccination strategy that also includes the medium- and high-risk population groups (Figure 10).

## Discussion

Although the development of an effective HIV vaccine is still years away, modeling studies such as this one are relevant and critical to preparing governments for adopting appropriate policies when a vaccine becomes available [8], [29] or as a vaccine candidate advances into later stages of clinical testing. Mathematical modeling and computer simulation are powerful tools for health policy evaluation, policy dialogue, and advocacy efforts [8]–[9], [30]–[31].

Spectrum was able to effectively model the Brazilian epidemic, producing projections of the number of new infections, AIDS deaths and people needing ARV based on various vaccine profiles and vaccination strategies. This exercise has shown that, even in a country like Brazil with a concentrated epidemic and ample availability of treatment and services for people with HIV and AIDS, a partially-effective vaccine could significantly reduce the number of new HIV infections and the future need for treatment, as well as the deaths caused by AIDS.

A unique aspect of this modeling application is that the epidemiological model was validated by comparing estimates of AIDS deaths for the period 1985 to 2007 with those reported by the vital registration system. This validation also included the rapid decline in AIDS deaths after the introduction of ART. It is possible that all AIDS deaths were not reported and that Spectrum has overestimated the AIDS deaths in Brazil. However, several studies have shown that the Brazilian Mortality System is quite reliable in terms of completeness and accuracy, as well as in relation to cause of death [32]–[33].

In the base projection, the annual number of new infections increases somewhat from 2010 to 2020 largely due to an increase among IDU, required to maintain constant prevalence after a period of declining prevalence among that group. A projection of a constant number of new HIV infections among IDU may be equally likely but, in either case, this assumption has little effect on the major conclusions. Also, the base projection without a vaccine shows a slight rise in the number of AIDS deaths. This is due to increasing mortality among those who have been on ART since the rapid scale-up of ART coverage in the 1990s.

Even though the model replicates the historical trends in Brazil, there is no guarantee that the model is correct in all aspects. With so many factors influencing model behavior it is likely that there are other sets of parameters and behavioral values that would fit the historical trends equally well. The impact of vaccines might be different with these other configurations. Advanced modeling techniques can be used to explore the impacts in the complete parameter space [34]–[35] but we have not done that here.

The impact analysis focuses on the period after 2020 when 80% coverage is reached and the impact of the vaccine is most significant. This relatively high coverage rate was deemed realistic by the project's steering committee - formed by Brazilian advisors with thorough understanding of the national epidemic - because of Brazil's relatively strong health infrastructure and successful coverage of existing health technologies, such as currently licensed vaccines and ARVs.

Efficacies of 40% and 70% were chose to represent a plausible range of likely vaccines that might be used in national programs. While a vaccine with efficacy greater than 70% would certainly be desirable, the difficulties to date in developing an effective vaccine indicates that the first vaccines to become available are not likely to provide perfect protection. Vaccines with efficacies below 30–40% are not likely to become approved for widespread use given the possibilities of risk compensation leading to perverse results. The recently completed RV144 vaccine trial indicated efficacy of around 30% [36]. These results are being used to guide future research rather than supporting immediate introduction of the tested vaccine.

A vaccine with 40% effectiveness given to 80% of the population would reduce susceptibility by 32% (40% times 80%), but the long-term effect is much larger, 72% from 2020 to 2050. The reason is that the reduction in susceptibility leads to fewer new infections which, in turn leads to lower prevalence. Thus in the future the susceptible population not only has reduced susceptibility to infection but also faces a reduced risk of encountering and infected partner. Over the long-term these secondary effects can be as large or larger than the reduction in susceptibility.

The analysis presented here has shown that vaccinating the adult population achieved the largest reduction of new HIV infections and deaths compared to other strategies. However, vaccinating specific population groups, such as those with higher-risk behavior, produced a greater impact with fewer vaccine doses required, which is important to note in resource-constrained environments. Through the use of targeted strategies, a significant proportion of the groups at increased risk for HIV can be reached with a smaller number of vaccinations than those required for vaccinating the adult population. For this reason, it is likely that a targeted vaccination strategy focusing on higher incidence groups would likely be more cost-effective, with a higher number of infections averted per vaccination. However, it is crucial that vaccination strategies for the adult population be designed to reach groups at greater risk, ensuring that these groups have equal access to the vaccine. Vaccinating only individuals at lower risk for HIV would significantly diminish the impact of the vaccine, compromising the goal of controlling the epidemic.

One concern with regards to implementing partially efficacious vaccines is the issue of behavioral disinhibition, which occurs when vaccinated individuals believe they are protected from HIV infection and therefore increase the behaviors that put them at greater risk of exposure to HIV [6]. Several studies have described how behavioral reversals could mitigate the gains from vaccination or even produce perverse outcomes where the use of the vaccine results in more infections [8], [37]–[39]. This study reinforces those findings. Even in a country with a concentrated epidemic, disinhibition will be a great challenge with the potential to negate the impact of a partially efficacious HIV vaccine. Efforts should be made to keep adherence to preventive measures at a high level, especially among populations at greater risk of infection, even when an HIV vaccine becomes available.

Although vaccines that partially prevent infection may produce a greater impact on the course of the epidemic, it is important to stress the potential benefits of a disease-modifying vaccine. This modeling exercise has demonstrated that such a vaccine could reduce AIDS deaths and also substantially decreases the demand for ARV immediately after introduction. A disease-modifying vaccine would also reduce infectiousness, further contributing to control the epidemic [40]–[42]. Without a reduction in infectiousness, disease-modifying vaccines would likely lead to more infections [40]–[41], [43]–[44] but fewer deaths [40], [44]. This study did not investigate the impact of a vaccine in either the absence of or reduction in ARV coverage because Brazil has a governmental policy to provide treatment to all in need.

The potential impact of even a moderate-efficacy vaccine as indicated in this study can also provide a powerful argument for the relevance of continued investment in research and development towards a vaccine as it presents evidence of how efforts to control HIV would dramatically benefit from it, even in the context of concentrated epidemics. For middle-income countries such as Brazil that have well established scientific and technological capacity, the results of this study, which highlight the relevance of a vaccine for the Brazilian epidemic, as well as others with same characteristics, should stimulate the implementation of more effective policies to promote and facilitate research in this field. Moreover, conclusions of the study related to the relative impact of vaccination strategies may prove particularly relevant in informing and accelerating discussions around vaccine introduction and delivery encouraging rapid adoption of a future product.
