# Correction: Modeling HIV Vaccines in Brazil: Assessing the Impact of a Future HIV Vaccine on Reducing New Infections, Mortality and Number of People Receiving ARV

**DOI:** 10.1371/annotation/4fd10061-5a1e-4164-833e-34943a74301c

**Published:** 2010-08-18

**Authors:** Maria Goretti P. Fonseca, Steven Forsythe, Alexandre Menezes, Shilpa Vuthoori, Cristina Possas, Valdiléa Gonçalves Veloso, Francisca de Fátima Lucena, John Stover

The sixth author's name was incomplete. The correct name is: Valdiléa Gonçalves Veloso. 

